# The Persistence of the Impact of COVID-19–Related Distress, Mood Inertia, and Loneliness on Mental Health During a Postlockdown Period in Germany: An Ecological Momentary Assessment Study

**DOI:** 10.2196/29419

**Published:** 2021-08-26

**Authors:** Matthias Haucke, Shuyan Liu, Stephan Heinzel

**Affiliations:** 1 Department of Psychiatry and Psychotherapy Charité – Universitätsmedizin Berlin Campus Charité Mitte Berlin Germany; 2 Department of Education and Psychology Clinical Psychology and Psychotherapy Freie Universität Berlin Berlin Germany

**Keywords:** COVID-19, outbreaks, epidemics, pandemics, psychological responses and emotional well-being, ecological momentary assessment, risk and protective factors, low incidence and restrictions

## Abstract

**Background:**

The first wave of the COVID-19 pandemic in early 2020 increased mental health problems globally. However, little is known about mental health problems during a low-incidence period of the pandemic without strict public health measures.

**Objective:**

We aim to investigate whether COVID-19–related risk factors for mental health problems persist beyond lockdown measures. We targeted a vulnerable population that is at risk of developing low mental health and assessed their daily dynamics of mood and emotion regulation after a strict lockdown.

**Methods:**

During a postlockdown period in Germany (between August 8, 2020, and November 1, 2020), we conducted an ecological momentary assessment with 131 participants who experienced at least mild COVID-19–related distress and loneliness. To estimate negative mood inertia, we built a lag-1 three-level autoregressive model.

**Results:**

We found that information exposure and active daily COVID-19 cases did not have an impact on negative mood amid a postlockdown period. However, there was a day-to-day carryover effect of negative mood. In addition, worrying about COVID-19, feeling restricted by COVID-19, and feeling lonely increased negative mood.

**Conclusions:**

The mental health of a vulnerable population is still challenged by COVID-19–related stressors after the lifting of a strict lockdown. This study highlights the need to protect mental health during postpandemic periods.

## Introduction

The COVID-19 pandemic and its associated socioeconomic consequences increased global mental health problems [1,2]. Negative mental health outcomes of the COVID-19 pandemic are associated with fear of becoming infected [3,4] and various mitigation strategies to curb the spread of COVID-19 (eg, curfews and restrictions to public life). These measures can disrupt regular routines, impair mood homeostasis [5-7], and impose economic hardship (eg, income loss and unemployment) [8], which can fuel anxiety, depression, and loneliness [9-12]. However, it is unclear whether these effects continue after lockdown measures have been eased. As variants emerge and cause sudden spikes in COVID-19 case numbers (eg, the B.1.1.7 variant in the United Kingdom in late 2020), fear of getting infected and/or another lockdown could persist. Moreover, after the pandemic and lockdown measures end, socioeconomic uncertainty remains [13]. Chronic psychological distress and social isolation are risk factors for developing mental disorders such as psychosis, substance abuse disorder, and affective disorder [14-17]. To investigate whether COVID-19–related stressors remain beyond lockdown measures, we set up an ecological momentary assessment (EMA) study in Germany during a postlockdown period. We focus on a group at high risk of poor mental health: those who experienced at least mild psychological distress and loneliness amid the COVID-19 pandemic. We expect a carryover effect of negative mood from one measurement to the next (mood inertia) and assume that COVID-19–related stressors (ie, momentary COVID-19–related worry, COVID-19 information seeking and perceived restriction, loneliness, and daily reported COVID-19 cases) result in an increase in momentary negative mood (Multimedia Appendix 1).

## Methods

### Study Design and Sampling

We conducted an EMA that involves repeated sampling of individuals’ current behaviors and experiences in real time and in their natural environments [18] during a postlockdown period (from August 8, 2020, to November 1, 2020) in Germany, when restrictions were lenient (eg, no private or public meeting restrictions, reopening of most leisure facilities, bars, and catering facilities; see Multimedia Appendix 1). EMA aims to minimize recall bias, maximize ecological validity, and approximate temporal causality (ie, Granger causality) and allows researchers to study microprocesses that influence behavior in real-world contexts [19]. Participants were recruited via online advertisements on universities’ websites, Twitter, and eBay classifieds. Participants had to fill in an online prequestionnaire on the Siuvo Intelligent Psychological Assessment Platform. After an initial contact via phone or email, we sent participants our study information, informed consent, and a QR code (to install a smartphone app) by mail.

We targeted vulnerable individuals who reported at least mild psychological distress and sometimes felt lonely amid the COVID-19 pandemic. We used the COVID-19 Peritraumatic Distress Index (CPDI [20]; cutoff score=28, indicating mild distress) questionnaire and the short-form version of the UCLA Loneliness Scale (ULS-8 [21]; cutoff score=16, indicating mild loneliness), respectively. Other inclusion criteria were being at least 18 years of age, not working night shifts, not currently infected with COVID-19, using an Android smartphone, and speaking fluent German. The CPDI was designed to evaluate changes in mental health status, cognitive skills, avoidance and compulsive behavior, physical symptoms, and loss of social functioning due to the COVID-19 pandemic. The questionnaire has been previously validated in a sample in Germany [20].

### Data Collection

We used a smartphone app called “movisensXS” (movisens GmbH), which was developed for research purposes. The app is compliant with the General Data Protection Regulation (European Union) and Berlin Data Protection Act (Berliner Datenschutzgesetz – BlnDSG). Participants completed a 20-minute baseline assessment, followed by 7 consecutive days in which they received 8 randomized prompts between 8 AM and 10 PM. The study procedure was approved by the Ethics Committees of Charité – Universitätsmedizin Berlin (ref: EA2/143/20) and Freie Universität Berlin (ref: 030/2020).

### Measurements

To quantify COVID-19–related distress, we measured worries about the COVID-19 pandemic, perceived restrictions due to the COVID-19 pandemic, COVID-19 information exposure, and feelings of loneliness. Finally, we measured respondents’ momentary negative mood (anxiety, depression, fatigue, stress, and unhappiness). All questions were measured on a visual analogue scale ranging from 0 (not at all) to 100 (very much). To account for the steady increase in active COVID-19 cases in Germany during the time of measurement [22], we included daily COVID-19 cases as a predictor in our analysis. Our smartphone study consisted of a sociodemographic assessment (ie, age, gender, years of education) and the EMA. The exact EMA items can be found in Multimedia Appendix 1 and online at [23].

### Statistical Analysis

All statistical analyses were conducted in R (version 3.5.3; R Foundation for Statistical Computing [24]). To consider the hierarchical data structure and autoregressive parameters, we performed model selection using autoregressive (AR) multilevel models with the dependent variable negative mood. We followed the approach by Haan-Rietdijk et al [25]; details about the model selection procedure can be found in Multimedia Appendix 1 and online at [23].

## Results

**Figure 1:**
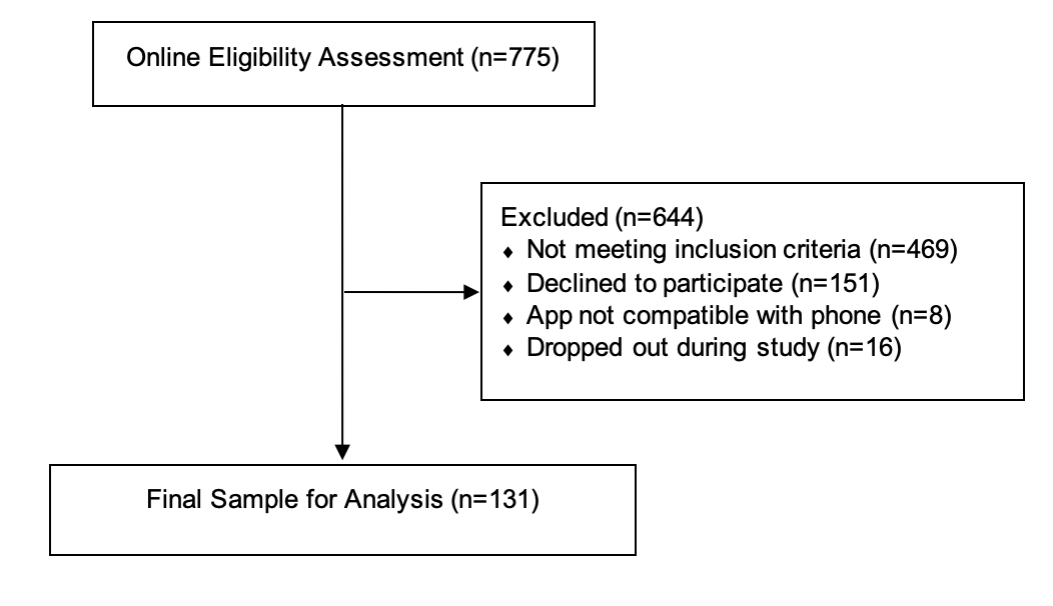
Recruitment flow.

We assessed 775 people for eligibility in an online questionnaire. The final sample size was 131 (18%; recruitment flow is shown in Figure 1 and sample characteristics are shown in Table 1; for power estimation, see Multimedia Appendix 1). No participant filled in less than 28 (50%) of the daily questionnaires, while 40 (<0.01%) of the total sent daily questionnaires were not answered by the participants.

**Table 1:** Demographics and sample characteristics.

Parameter	Values
COVID-19 Peritraumatic Distress Index score, mean (SD)	48.42 (16.31)
UCLA Loneliness Scale score, mean (SD)	22 (4.03)
Education (in years), mean (SD)	15.08 (3.66)
Age (in years), mean (SD)	31.62 (10.76)
Gender, n (%)	Male: 49 (37); female: 82 (63)

We used a lag-1 three-level AR model, which allows us to separate the variance of negative mood scores into variance at the person level (level 3), variance at the day level (level 2), and variance at the questionnaire level (level 1). We created two lagged variables, a within-day centered predictor at questionnaire level and a within-person centered lagged predictor at the day level. The very first beep of each day (ie, the time period between the previous day’s beep and next day’s beep) was excluded from the analysis to remove possible unexplained carryover effects resulting from the night (eg, lack of sleep). This model includes mood inertias, COVID-19 worries, COVID-19 information seeking, perceived restrictions, and loneliness during the last hour, as well as daily active COVID-19 cases as random effects. The momentary negative mood score was built by averaging momentary feelings of fatigue, anxiety, depression, unhappiness, and stress. A graphical check indicated a positive skew of negative mood; therefore, we performed a square root transformation on this variable. The analysis script can be found online at [23].

**Figure 2:**
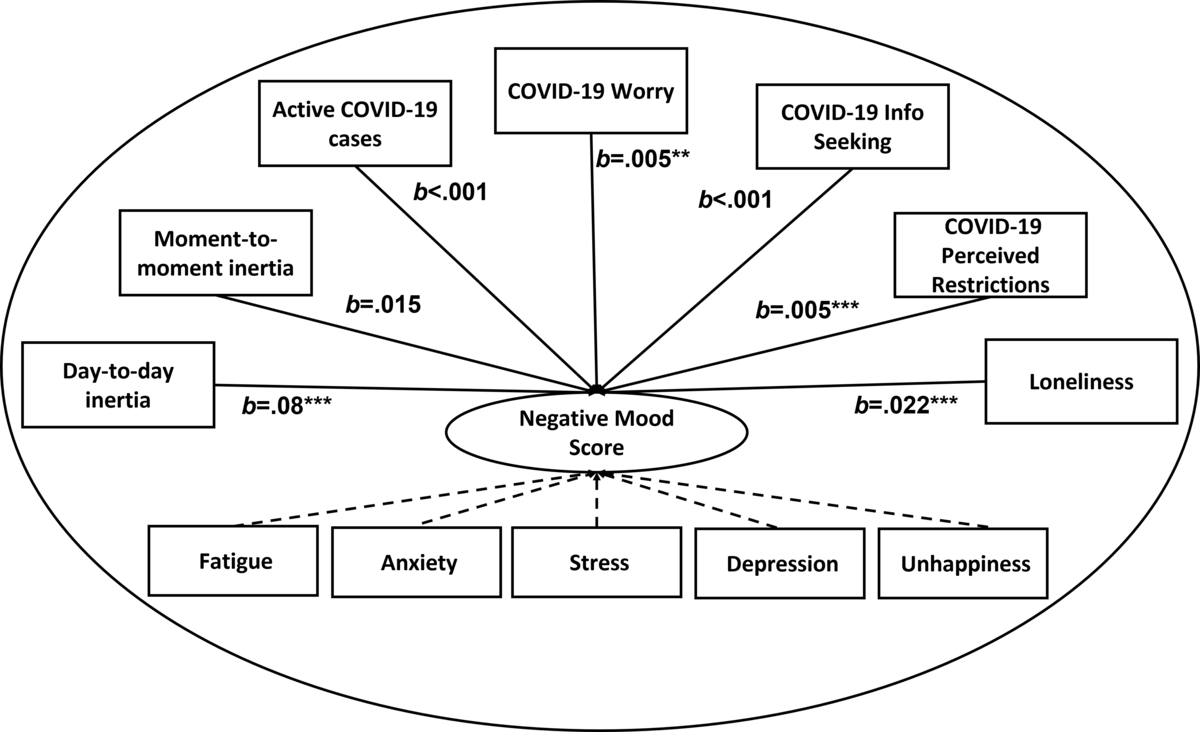
Loneliness, COVID-19 worries, feelings of restriction, and day-to-day mood inertia increased negative mood. Moment-to-moment mood inertia, active COVID-19 cases, and COVID-19 information seeking did not increase negative mood. *P<.05, **P<.01, ***P<.0001 (two-tailed). N=131.

We found that loneliness (*b*=.022, t_3713.83_=18.68, *P*<.001), COVID-19 perceived restriction (*b*=.005, t_129.84_=3.65, *P*<.001), COVID-19–related worry (*b*=.005, t_132.74_=2.87, *P*=.001), and day-to-day mood inertia (*b*=.078, t_134.58_=3.96, *P*=.001) increased negative mood scores. Active daily COVID-19 case numbers (*b*<.001, t_92.17_=–0.27, *P*=.87), COVID-19–related information seeking (*b*<.001, t_88.41_=0.73, *P*=.47), and moment-to-moment inertia (*b*=.015, t_42.19_=0.17, *P*=.87) did not increase negative mood scores (see Figure 2).

## Discussion

### Principal Findings

We found that negative effects of the COVID-19 pandemic on mental health outlast lockdown measures. In line with findings from the first COVID-19 wave [8,26-30], we found that loneliness, worrying about COVID-19, and perceived restriction increased negative mood during a postlockdown period. Similar to the Ebola pandemic [31], possible reasons for the lasting effect of the COVID-19 pandemic might be worries about the negative economic consequences, concern about resurgence of the virus, struggles to rebuild social networks, and/or deliberately withdrawing from social contacts to avoid infection.

Furthermore, we found a negative carryover effect of mood between days (mood inertia), indicating dysfunctional mood regulation. Restrictive policies during the COVID-19 pandemic can impact mental health, possibly due to impaired mood homeostasis (ie, failure to positively regulate mood via mood-modifying activities) [7]. Importantly, our results show that even when the acute threat and restrictive measures are less pronounced, negative daily mood inertia remains.

Neither COVID-19 information seeking nor active COVID-19 cases increased negative mood. This contrasts with previous findings from lockdown periods [6,32]. For example, an EMA study during the first lockdown in Germany and Austria reported increased perceived COVID-19–related restrictions that were positively associated with increased daily news consumption, especially in individuals living alone [32]. In addition, an EMA study conducted in New Jersey in the United States between April 24 and May 26, 2020, showed that undergraduates felt more anxious about COVID-19 on days when the number of new cases and deaths due to COVID-19 were higher [6]. Our opposing finding might be caused by the relatively low domestic case numbers and associated news during the postlockdown period. Moreover, negative COVID-19 news might have less impact on mood over the course of the pandemic as people get accustomed to it.

### Limitations

We did not make explicit comparisons to participant status before the COVID-19 outbreak or to a control group, which limits generalizability to other populations. Furthermore, we did not measure adaptability, which has been associated with positive mood (eg, optimism and satisfaction) [33]. Finally, we did not assess the nature of COVID-19 worries and reasons for feeling restricted.

### Conclusions

Even if cases are low and lockdown policies are lenient, mental health is still challenged by COVID-19–related stressors. Although information exposure to COVID-19 and daily COVID-19 cases had no impact on mood, we found a day-to-day carryover effect of negative mood. Moreover, COVID-19–related restriction, worry about COVID-19, and loneliness increased negative mood. Thus, the negative impact of the COVID-19 pandemic on mental health outlasts lockdown measures and mental health challenges will likely continue after the pandemic.

## Appendix

Multimedia Appendix 1 Supplementary materials.

## Abbreviations

AR: autoregressive
CPDI: COVID-19 Peritraumatic Distress Index
EMA: ecological momentary assessment
ULS-8: short-form UCLA Loneliness Scale
