# Supplementary material for: The Persistence of the Impact of COVID-19–Related Distress, Mood Inertia, and Loneliness on Mental Health During a Postlockdown Period in Germany: An Ecological Momentary Assessment Study
Source: JMIR Ment Health. 2021 Aug 26;8(8):e29419. doi: 10.2196/29419 (PMC8396535; doi:10.2196/29419)
Supplement: Multimedia Appendix 1 [file mental_v8i8e29419_app1.docx]

**Supplementary Material A: Further Background Information**

The coronavirus disease 2019 (COVID-19) is continuing to spread around the world. The World Health Organization (WHO) reports, as of May 2021, 164,523,894 confirmed cases and 3,412,032 deaths [1]. To mitigate the spread of the COVID-19 virus, most countries enforced lockdown measures, including social restrictions, travel bans, stay-at-home orders, and business shutdown. These measures had major impact on the mental health of the general population and may have profound and long-lasting consequences [2]. Negative mental health outcomes of the COVID-19 pandemic are associated with fear of becoming infected [3, 4]. A survey during the initial outbreak of the COVID-19 outbreak in China found that 53.8% of respondents rated the psychological impact of the outbreak as moderate or severe [5]. Previous studies during the Ebola outbreak have shown that the fear of the virus is associated with the experience of intense distress and pose a risk factor for long-term mental and psychosocial problems, such as anxiety, mood disorder as well as acute stress reactions [6]. Moreover, public health responses (i.e., closing off business and prohibiting physical contact) disrupt daily routines, impair mood homeostasis [7-9] and impose economic hardship (e.g., income loss and unemployment) [10] which, in turn, can increase anxiety, depression and loneliness and distress [11-14]. Chronic psychological distress and social isolation are risk factors for developing mental disorders, such as psychosis, substance abuse disorder and affective disorder [15-18]

We measured three COVID-19 related stressors: firstly, feelings of restriction in everyday life, secondly, seeking information about the pandemic, and thirdly, worrying about the pandemic and its impact on one’s life. Worries about the COVID-19 related economic downfall, and the possible health impact on oneself or others can increase psychological distress [10, 19]. In addition, anxiety, psychological distress, and worries increase with the common public health measure of physical distancing [20, 21]. Moreover, if people are staying at home more often, they will more likely be exposed to pandemic related digital and social media information, which in turn increases anxiety and stress [8, 22]. To conclude, there are three stressors central to the pandemic: COVID-19 related feelings of restriction, information seeking and worry.

Social restrictions and other lockdown measures help limit the spread of COVID-19; however, during the first wave of the pandemic, these measures have also led to an increase in feelings of loneliness [10, 23]. Loneliness can be defined as an aversive state resulting from a discrepancy between an individual’s desired and realized social relationships [24]. Loneliness has severe impacts one people’s health, increasing cardiovascular disease and immune dysfunction, depression, anxiety, and suicidal ideation [25].

In Germany, there is an increased demand in psychological counseling because of the COVID-19 pandemic and lockdown measures [26]. This increase indicates heightened loneliness, anxiety, and even suicidal ideation and is more pronounced in German federal states that implemented stricter measures [26]. It is unclear whether the negative effects of COVID-19 will continue after lockdown measures have been eased. As variants occur with a sudden spike in COVID-19 case numbers (e.g., India in April 2021 [27]), fear of getting infected and another lockdown could persist. Moreover, after the pandemic and lockdown measures are over, socio-economic uncertainty remains [28]. To investigate whether COVID-19 related stressors remain beyond lockdown measures, we set up an ecological momentary assessment (EMA) study in Germany during a post-lockdown phase.

**Supplementary Material B: Lockdown measures**

Summary of measures to counteract the pandemic in Germany between August 2020 and 01 November 2020 (https://www.deutschland.de/en/news/german-federal-government-informs-about-the-corona-crisis)

1. Nationwide, a distance of at least 1.5 meters must be maintained, hygiene rules must be observed, and masks must be worn in shops and on public transport. There was no general restriction on public meetings.

2. Institutions and leisure facilities (i.e., theatres, concert halls, cinemas, and fitness studios) opened.

3. Sports and recreational activities indoor and outdoor were permitted.

4. Restaurants, bars, pubs, cafés, and other catering establishments opened.

All above-mentioned policies must act in strict compliance with hygiene and infection control regulations.

5. Governmental financial aid for those that suffered economic losses during the time of the pandemic.

**Supplementary Material C: Power estimation**

Based on within-subject reliability of assessments performed on a smartphone, power analyses are recommended based on even smaller effect sizes [29]. Conventional power analyses for multiple regression models indicate a sample size of N = 115 for detecting small effect sizes (f = .05) using predictor sets of up to 7 variables. Considering a drop-out rate of 25%, a total size of 144 is required.

**Supplementary Material D: Modell building process**

To determine whether a two-level and three-level lag- 1 autoregressive (AR1) models is more appropriate for our data, we followed a model selection procedure using the Akaike Information Criterion [30] described by de Haan-Rieddijk and colleagues [31].

First, we build the most basic, empty (or intercepts-only) two-level model (**2-l. empty**), which accounts for the fact that we have measurements within persons, but which does not include an autoregressive parameter. Then, we build the two-level AR(1) model, in which each affect score is regressed on the immediately preceding affect score of that person. For this two-level AR (1) model, we build a model in with a correlation between random effects (**2-l. AR(1)**), and a constrained model where the correlation is fixed at zero (**2-l. AR(1) no corr**.), which is achieved by explicitly separating the random intercept and the random predictor.

We build a three-level AR(1) model (**3-l. empty**), which accounts for the fact that the beeps (each questionnaire) are nested within persons, and for the multi-day structure of the data. This results in distinct inertia parameters for the carry-over from day to day and from moment to moment. Thus, we can partition the variance in the affect scores into variance at the person level (level 3), variance at the day level (level 2), and variance at the beep level (level 1). Again, two models were constructed, one with a correlation between random effects (**3-l. AR(1)**), and one constrained model where the correlation is fixed at zero (**3-l. AR(1) no corr**.).

We created two lagged variables, a within-day centered predictor at questionnaire level, and a within-person centered lagged predictor at the day level. The first day and the first questionnaire of each day were excluded from the analysis of the three-level model to exclude carry over-effects resulting from processes prior to the study and from the night. To compare the AIC of the AR(1) models, the two-level AR(1) models were refitted to the smallest of the data, to those cases that could also be used in the three-level AR(1) model with day-level inertia. Based on the AIC, we selected the model **3-l. AR(1) no corr** (see **Table 1**).

| Model | AIC |
| --- | --- |
| 2-l. empty | 13376 |
| 3-l. empty | 12956 |
| 2-l. AR(1) no corr. | 13310 |
| 2-l. AR(1) | 13312 |
| **3-l AR(1) no corr** | **12895** |
| 3-l AR(1) | 12898 |

**Table 1.** The models are estimated on the smallest suitable subset of data to ensure equal sample size. The bold model indicates the model that was selected for the final analysis.

**Adding more predictors:**

Finally, we added the predictors COVID-19 worries, information seeking, perceived restrictions, and loneliness during the last hour as well as COVID-19 case numbers as random effects. The outcome variable momentary negative mood score was built by averaging momentary feelings of fatigue, anxiety, depression, unhappiness, and stress.

**Supplementary Material E: EMA Items**

**Mood Items**

In diesem Moment...

In this moment….

Unhappy:

.. fühle ich mich unglücklich.

.. I feel unhappy.

Fatigue:

..fühle ich mich müde.

..I feel tired.

Stress:

…habe ich das Gefühl, unter Stress zu stehen.

..I feel stressed.

Anxiety:

...fühle ich mich ängstlich.

.. I feel anxious.

Depression:

.. fühle ich mich niedergeschlagen.

.. I feel depressed.

**COVID-19 and loneliness Items**

In der letzten Stunde...

During the last hour…

Corona restriction:

...in welchem Ausmaß haben Sie sich durch die Pandemie in Ihrem Alltag eingeschränkt gefühlt?

… to which extent did you feel constrained by the pandemic in your everyday life?

Corona worry:

...in welchem Ausmaß haben Sie darüber nachgedacht wie die Pandemie Ihre persönliche Lebenssituation beeinflusst?

.. to which extent did you worry about how the pandemic affects your personal situation?

Corona Information Seeking:

...in welchen Ausmaß haben Sie Informationen zur Corona Pandemie gelesen/gesehen?

.. to which extent did you read/see Information about the Corona pandemic?

Loneliness:

.. wie sehr fühlten Sie sich einsam?

.. how lonely did you feel?

**Supplementary Material F: Supplementary References**

1. World Health Organization (WHO). WHO coronavirus disease (COVID-19) dashboard. Accessed May 21, 2021. https://covid19.who.int/

2. Brooks SK, Webster RK, Smith LE, Woodland L, Wessely S, Greenberg N, et al. The Psychological Impact of Quarantine and How To Reduce it: Rapid Review of the Evidence. The Lancet 2020 Mar 14;395(10227):912-20. doi: 10.1016/S0140-6736(20)30460-8

3. Nelson BW, Pettitt AK, Flannery JE, Allen NB. Rapid Assessment of Psychological and Epidemiological Correlates of COVID-19 Concern, Financial Strain, and Health-Related Behavior Change in a Large Online Sample [Internet]. PsyArXiv; 2020. [doi: 10.31234/osf.io/jftze]

4. Cao W, Fang Z, Hou G, Han M, Xu X, Dong J, et al. The Psychological Impact of the COVID-19 Epidemic on College Students in China. Psychiatry Res 2020 May;287:112934. doi: 10.1016/j.psychres.2020.112934

5. Wang C, Pan R, Wan X, Tan Y, Xu L, Ho CS, et al. Immediate Psychological Responses and Associated Factors during the Initial Stage of the 2019 Coronavirus Disease (COVID-19) Epidemic among the General Population in China. Int J Environ Res Public Health. 2020 Mar 6;17(5):1729. doi: 10.3390/ijerph17051729.

6. Shah K, Kamrai D, Mekala H, Mann B, Desai K, Patel RS. Focus on Mental Health During the Coronavirus (COVID-19) Pandemic: Applying Learnings from the Past Outbreaks. Cureus 2020 Mar 25;12(3):e7405. doi: 10.7759/cureus.7405.

7. Saha K, Torous J, Caine ED, De Choudhury M. Psychosocial Effects of the COVID-19 Pandemic: Large-scale Quasi-Experimental Study on Social Media. J Med Internet Res 2020 Nov 24;22(11):e22600. doi: 10.2196/22600

8. Kleiman EM, Yeager AL, Grove JL, Kellerman JK, Kim JS. Real-time Mental Health Impact of the COVID-19 Pandemic on College Students: Ecological Momentary Assessment Study. JMIR Mental Health. 2020;7(12):e24815.

9. Taquet M, Quoidbach J, Fried EI, Goodwin GM. Mood Homeostasis Before and During the Coronavirus Disease 2019 (COVID-19) Lockdown Among Students in the Netherlands. JAMA Psychiatry 2021 Jan 01;78(1):110-112. doi: 10.1001/jamapsychiatry.2020.2389

10. Liu S, Heinzel S, Haucke MN, Heinz A. Increased Psychological Distress, Loneliness, and Unemployment in the Spread of COVID-19 over 6 Months in Germany. Medicina (Kaunas) 2021 Jan 09;57(1):53. doi:10.3390/medicina57010053

11. Huckins JF, daSilva AW, Wang W, Hedlund E, Rogers C, Nepal SK, et al. Mental Health and Behavior of College Students During the Early Phases of the COVID-19 Pandemic: Longitudinal Smartphone and Ecological Momentary Assessment Study. J Med Internet Res 2020 Jun 17;22(6):e20185. doi: 10.2196/20185

12. Rajkumar RP. COVID-19 and mental health: A review of the existing literature. Asian J Psychiatr 2020 Aug;52:102066. doi: 10.1016/j.ajp.2020.102066

13. Munblit D, Warner J, Tudor-Williams G, Petrou L, Cheung K. Excessive Media Consumption About COVID-19 is Associated With Increased State Anxiety: Outcomes of a Large Online Survey in Russia. J Med Internet Res. 2020 Sep 11;22(9):e20955. doi: 10.2196/20955.

14. Zhang W, Yang X, Zhao J, Yang F, Jia Y, Cui C, et al. Depression and Psychological-Behavioral Responses Among the General Public in China During the Early Stages of the COVID-19 Pandemic: Survey Study. J Med Internet Res. 2020 Sep 4;22(9):e22227. doi: 10.2196/22227.

15. Deng W, Cheung S, Tsao S, Wang X, Tiwari A. Telomerase Activity and its Association with Psychological Stress, Mental Disorders, Lifestyle Factors and Interventions: A Systematic Review. Psychoneuroendocrinology. 2016 Feb;64:150-63. doi: 10.1016/j.psyneuen.2015.11.017. Epub 2015 Nov 25.

16. Heinz A, Deserno L, Reininghaus U. Urbanicity, Social Adversity and Psychosis. World Psychiatry. 2013 Oct;12(3):187-97. doi: 10.1002/wps.20056.

17. Narita Z, Stickley A, DeVylder J. Loneliness and Psychotic Experiences in a General Population Sample. Schizophr Res. 2020 Apr;218:146-150. doi: 10.1016/j.schres.2020.01.018. Epub 2020 Jan 31.

18. Beutel ME, Klein EM, Brähler E, Reiner I, Jünger C, Michal M, et al. Loneliness in the General Population: Prevalence, Determinants and Relations to Mental Health. BMC Psychiatry. 2017 Mar 20;17(1):97. doi: 10.1186/s12888-017-1262-x.

19. Rauschenberg C, Schick A, Goetzl C, Roehr S, Riedel-Heller SG, Koppe G, et al. Social Isolation, Mental Health and Use of Digital Interventions in Youth During the COVID-19 Pandemic: A Nationally Representative Survey. Eur Psychiatry. 2021 Mar 9;64(1):e20. doi: 10.1192/j.eurpsy.2021.17.

20. Li LZ, Wang S. Prevalence and Predictors of General Psychiatric Disorders and Loneliness During COVID-19 in the United Kingdom. Psychiatry Res. 2020 Sep;291:113267. doi: 10.1016/j.psychres.2020.113267. Epub 2020 Jun 30.

21. Galea S, Merchant RM, Lurie N. The Mental Health Consequences of COVID-19 and Physical Distancing: The Need for Prevention and Early Intervention. JAMA Intern Med 2020 Jun 01;180(6):817-818. doi: 10.1001/jamainternmed.2020.1562

22. Amsalem D, Dixon LB, Neria Y. The Coronavirus Disease 2019 (COVID-19) Outbreak and Mental Health: Current Risks and Recommended Actions. JAMA Psychiatry. 2021 Jan 1;78(1):9-10. doi: 10.1001/jamapsychiatry.2020.1730.

23. Hwang T-J, Rabheru K, Peisah C, Reichman W, Ikeda M. Loneliness and Social Isolation During the COVID-19 Pandemic. Int Psychogeriatr. 2020 Oct;32(10):1217-1220. doi: 10.1017/S1041610220000988.

24. Cacioppo JT, Cacioppo S. Loneliness in the Modern Age: An Evolutionary Theory of Loneliness (ETL). Advances in experimental social psychology: Elsevier; 2018. p. 127-97. Doi: 10.1016/bs.aesp.2018.03.003

25. Singer C. Health effects of social isolation and loneliness. Journal of Aging life care. 2018;28(1):4-8.

26. Armbruster S, Klotzbücher V. Lost in lockdown? COVID-19, social distancing, and mental health in Germany. Diskussionsbeiträge. 2020; 2020-04.

27. Thiagarajan K. Why is India Having a Covid-19 Surge? BMJ. 2021 Apr 30;373:n1124. doi: 10.1136/bmj.n1124.

28. Nicola M., Alsafi Z., Kerwan A., et al. The Socio-Economic Implications of the Coronavirus Pandemic (COVID-19): A Review. Int J Surg. 2020 Jun;78:185-193. doi: 10.1016/j.ijsu.2020.04.018.

29. Sliwinski MJ, Mogle JA, Hyun J, Munoz E, Smyth JM, Lipton RB. Reliability and Validity of Ambulatory Cognitive Assessments. Assessment. 2018 Jan;25(1):14-30. doi: 10.1177/1073191116643164.

30. Akaike H. A New Look at the Statistical Model Identification. IEEE Transactions on Automatic Control. 1974 Dec; 19(6):716-723. doi: 10.1109/TAC.1974.1100705.

31. de Haan-Rietdijk S, Kuppens P, Hamaker EL. What's in a Day? A Guide to Decomposing the Variance in Intensive Longitudinal Data. Front Psychol. 2016 Jun 14;7:891. doi: 10.3389/fpsyg.2016.00891.
